# Characterization of a secreted macrophage migration inhibitory factor homologue of the parasitic nematode *Haemonchus Contortus* acting at the parasite-host cell interface

**DOI:** 10.18632/oncotarget.16675

**Published:** 2017-03-29

**Authors:** Yujian Wang, MingMin Lu, Shuai Wang, Muhammad Ehsan, RuoFeng Yan, XiaoKai Song, LiXin Xu, XiangRui Li

**Affiliations:** ^1^ College of Veterinary Medicine, Nanjing Agricultural University, Nanjing, PR China

**Keywords:** Haemonchus Contortus, mammalian migration inhibitory factor, monocyte, immunomodulation, Immunology and Microbiology Section, Immune response, Immunity

## Abstract

Modulation and suppression of the immune response of the host by nematode parasites have been reported extensively and the migration inhibitory factor (MIF) is identified as one of the major immunomodulator. In the present study, we cloned and produced recombinant MIF protein from the small ruminant’s nematode parasite *Haemonchus contortus* (rHCMIF-1), and investigated its immunomodulatory effects on goat monocyte. Enzymatic assays indicated that rHCMIF-1 possessed tautomerase activity. Immunohistochemical test demonstrated that the native HCMIF-1 protein was predominantly localized at the body surface and internal surface of the worm’s gut. We demonstrated that rHCMIF-1 could be distinguished by antisera from goats experimentally infected with *H. contortus* and could bind by goat monocytes. The immunomodulatory effects of HCMIF-1 on cytokine secretion, MHC molecule expression, NO production and phagocytosis were observed by co-incubation of rHCMIF-1 with goat monocytes. The results showed that the interaction of rHCMIF-1 decreased the production of TNF-α, IL-1β and IL-12p40, where as, it significantly increased the secretion of IL-10 and TGF β in goat monocytes. After rHCMIF-1 exposure, the expression of MHC-II on goat monocytes was inhibited. Moreover, rHCMIF-1 could down-regulate the LPS induced NO production of goat monocytes. Phagocytotic assay by FITC-dextran internalization showed that rHCMIF-1 could inhibit the phagocytosis of goat monocytes. Our findings provided potential targetas immunoregulator, and will be helpful to elucidate the molecular basis of host–parasite interactions and search for new potential protein as vaccine and drug target candidate.

## INTRODUCTION

*Haemonchus contortus (H. contortus)* is one of the most economically important parasites of small ruminants worldwide. Infection can lead to anaemia, loss of condition and death of the host, especially lambs [1, 2]. Highly complex and specialized host-parasite interactions are involved in the evasion of host immune response by nematode parasites that help in their prolonged survival with in the mammalian hosts [3, 4].To survive in the face of the host's immune system, the parasitic nematodes produce an array of molecules, located on the cuticle surface and/or being released as excretory secretory proteins (ESP), during host-parasite interface [5, 6]. Molecules expressed and secreted by the nematode that might modulate host immune responses include proteases, protease inhibitors, antioxidants and orthologs of host cytokines and their receptors [7].

Mammalian migration inhibitory factor (MIF) was among the first immune system cytokines to be discovered as a soluble factor released from activated lymphocytes that inhibited random migration of macrophages [8, 9]. The function of macrophage MIF has endured, although MIF is now recognized as being highly pleiotropic in regulation of innate and adaptive immune cell populations, as well as cells of the neuroendocrine system [10, 11]. Equally, MIF is notable for its production by a wide range of cell types, locally by macrophages [12], T cells [10], and eosinophils [13] as well as systemically by the anterior pituitary gland [14]. Lacking an N-terminal signal sequence sufficient for classical secretion via the ER and Golgi pathway, mammalian MIF is secreted by a leaderless, nonconventional pathway [15]. In the context of the mammalian immune system, MIF has been ascribed multiple functions, including roles in pathogenesis of septic shock [16], rheumatoid arthritis [17], inflammatory bowel disease and tumor metastasis [18]. Although the molecular mechanisms by which MIF mediates its actions at the cellular level remain only partially understood, one pathway that has been defined clearly is the human MIF (hMIF), a ligand for the CD74-CD44 receptor complex at the surface of target cells [19, 20]. MIF is unique in combining cell-stimulatory activity (cytokine) with two enzymatic activities within a relatively small protein [21], namely a tautomerase, which is dependent on Pro-2 [22], and an oxidoreductase dependent on cysteine residues 57 and 60, forming a thioredoxin-like motif [23]. A homolog of human MIF is produced by several parasite species belonging to both protozoans and nematodes [24]. Among nematodes, MIF have been reported in free living nematode *Caenorhabditis elegans* and parasitic nematodes such as *Trichuris sp*., *Onchocerca volvulus*, *Brugia pahangi*, *Strongolyidis stercoralis*, *Ancyclostoma sp*. and *Schistosoma sp*. [24].

In the present study, we cloned a MIF gene from *H. contortus*, produced the recombinant protein and analyzed its immune modulatory activity. We observed that the recombinant MIF from *H. contortus* (rHCMIF-1) significantly modulated goat monocyte function in multiple aspects.

## RESULTS

### Cloning and sequence analysis of HCMIF-1

**Figure 1:**
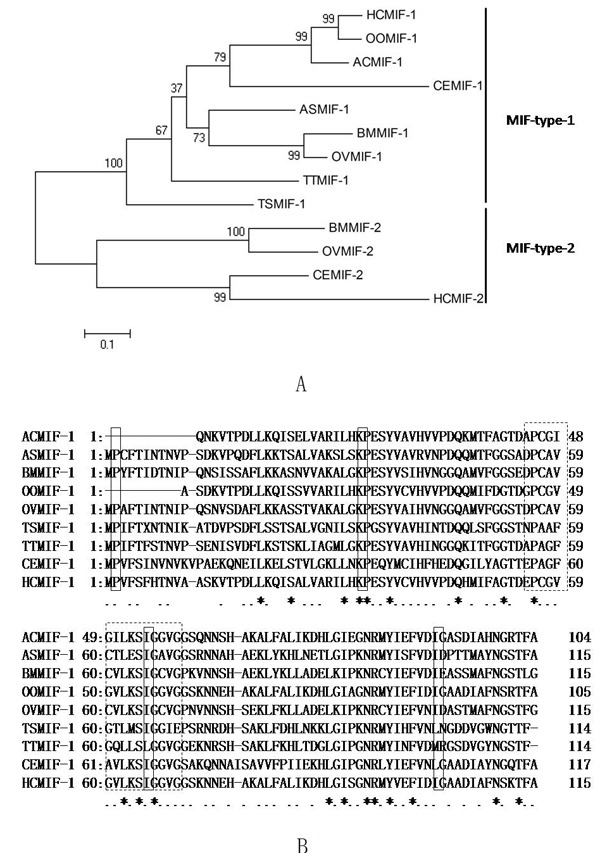
Relationship between HCMIF-1and other nematode MIF orthologues **A**. The neighbour joining tree was bootstrapped 1000 times using Clustal X and viewed with Tree View. Each node is annotated with a figure indicating the degree of bootstrap support for each branch. Abbreviations and accession numbers are as follows: TTMIF-1 of *Trichuris trichiura* (GenBank accession number: CAB46355); TSMIF-1 of *Trichinella spiralis* (GenBank accession number: CAB46354)]; ASMIF-1 of *Ascaris suum* (GenBank accession number: BAD24819)]; BMMIF-1 of *Brugia malayi* (GenBank accession number: AAB60943)]; BMMIF-2 of *B. malayi* (GenBank accession number: AAF91074); OOMIF-1 of *Ostertagia ostertagi* (GenBank accession number: BQ457911); HCMIF-1 of *Haemonchus contortus* (GenBank accession number: CDJ88729.1); HCMIF-2 of *H. contortus* (GenBank accession number: CB015598); OVMIF-1 of *Onchocerca volvulus* (GenBank accession number: AAK66563); OVMIF-2 of *O. volvulus* (GenBank accession number: AAK66564); ACMIF-1 of *Ancylostoma caninum* (GenBank accession number: AW626839); CEMIF-1 of *Caenorhabditis elegans* (NP_499536)]; CEMIF-2 of *C. elegans* (NP_506003)]. **B**. Amino acid alignment of HCMIF-1 and other MIF-1-type orthologues. Annotation is as described for Panel a. ‘Invariant’ residues of the MIF active site are boxed in solid pattern. Prosite MIF Pattern (Accession numberPS01158) [DE]-P- [CLV]- [APT]-x(3)- [LIVM]-x-S- [IS]- [GT]-x- [LIVM]- [GST] is shown as a dotted box.

While searching the online database, a MIF homologue was identified and cloned from *H. contortus*, designated HCMIF-1.It contains 115 amino acid residues with a predicted molecular mass of 12.3kDa and an isoelectric point of 7.39.The amino acid analysis using the SignalP program did not reveal the presence of a signal peptide. When the relationship between HCMIF-1 and selected orthologues from other nematode species was examined by neighbour joining analysis, HCMIF-1 clustered with MIF-1-type [24] rather than MIF-2-type orthologues (Figure 1a). Of the ‘invariant’ residues thought to be associated with tautomerase activity and the MIF substrate molecule-interaction site [24], HCMIF-1 possessed the N-terminal catalytic proline (Pro1, Figure 1b) which is exposed following post-translational cleavage of the initiating methionine [21], as well as conserved residues Lys32 and Ile64, but not Val106. The substitution of isoleucine for valine at position106 is common to each MIF-1-type sequence from Clade Vnematodes (Figure 1b). Clade I nematodes have valine at this residue; however each Clade III nematode has methionine at this position (Figure 1b). In addition, Prosite MIF Pattern (Accession number PS01158) [DE]-P- [CLV]- [APT]-x(3)- [LIVM]-x-S- [IS]- [GT]-x- [LIVM]- [GST] is only partly represented in HCMIF-1 as the motif ‘^54^EPCGVGVLKSIGGVG^68^’ where the residues that are not represented by the Prosite pattern are underlined (Figure 1b).

### Expression and purification of rHCMIF-1

**Figure 2:**
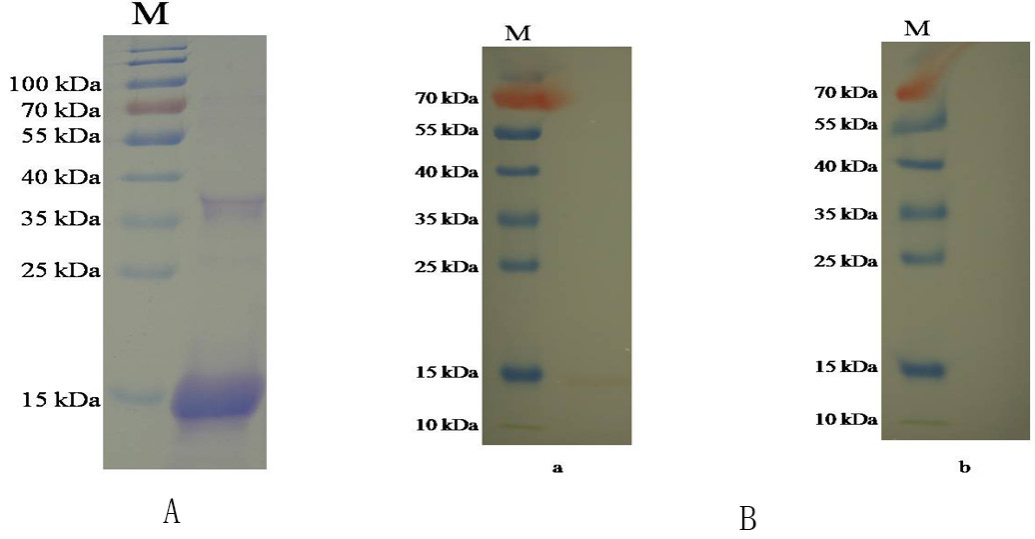
Purification and western blot of rHCMIF-1 **A**. Purified rHCMIF-1were resolved by SDS-PAGE on 12% of polyacrylamide gel and stained with Coomassie brilliant blue R250. **B**. Western blot analysis of Purified rHCMIF-1. Proteins are recognized by sera from goats experimentally infected with *H. contortus* as primary antibody (a) and normal goat sera (b) as control.

The gene encoding HCMIF-1 was ligated into the bacterial expression vector pET28a, and the recombinant was successfully expressed as a double His 6 tagged fusion protein with an expected size of 15 kDa (Figure 2a). The rHCMIF-1 was expressed in a soluble form and then purified by affinity chromatography using the His·Bind^®^128 Resin Chromatography kit (Novagen) according to the manufacturer's instructions. The purity of rHCMIF-1 was more than 95% as estimated by SDS-PAGE analysis.

### Western blot analysis

To determine whether the HCMIF-1 protein was interacted to host immune system, serum from goats experimentally infected with *H. contortus* was used as primary antibody to react with rHCMIF-1.Western blot analysis showed that the antiserum was able to recognize rHCMIF-1 (Figure 2b), suggesting that HCMIF-1 could interact with host immune system during parasitic process.

### Enzymatic activity

rHCMIF-1, expressed as soluble protein in a bacterial expression system, displayed dopachrome tautomerase activity (Table 1), convert both the substrates *p*-hydroxyphenylpyruvate and L-dopamethylester.

**Table 1:** Tautomerase activity of HCMIF-1

Substrate	Specific activity (μmol/min/mg protein) mean±standard deviation
*p*-hydroxyphenylpyruvate	236.34±5.66
L-dopa methyl ester	592.47±47.64

### Immunolocalization of HCMIF-1

**Figure 3:**
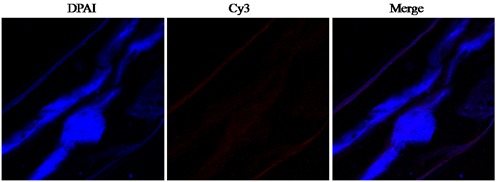
Immunohistochemical localization of HCMIF-1 protein in cryostal section of H. contortus.HCMIF-1 protein was detected by the indirect immunofluorescence method using second antibody Cy3 labeled goat anti-rat IgG (ab6953, Abcam). The section was counter stained with DAPI to show DNA.

A section through a partial body length of an adult female worm was shown in Figure 3. HCMIF-1 and DNA fluorescedred and blue, respectively. The antibody eluted from rHCMIF-1 bound predominantly to the body surface and internal surface of the parasite's gut (Figure 3) and no labeling was observed in control experiments.

### Binding of rHCMIF-1 to goat monocytes

**Figure 4:**
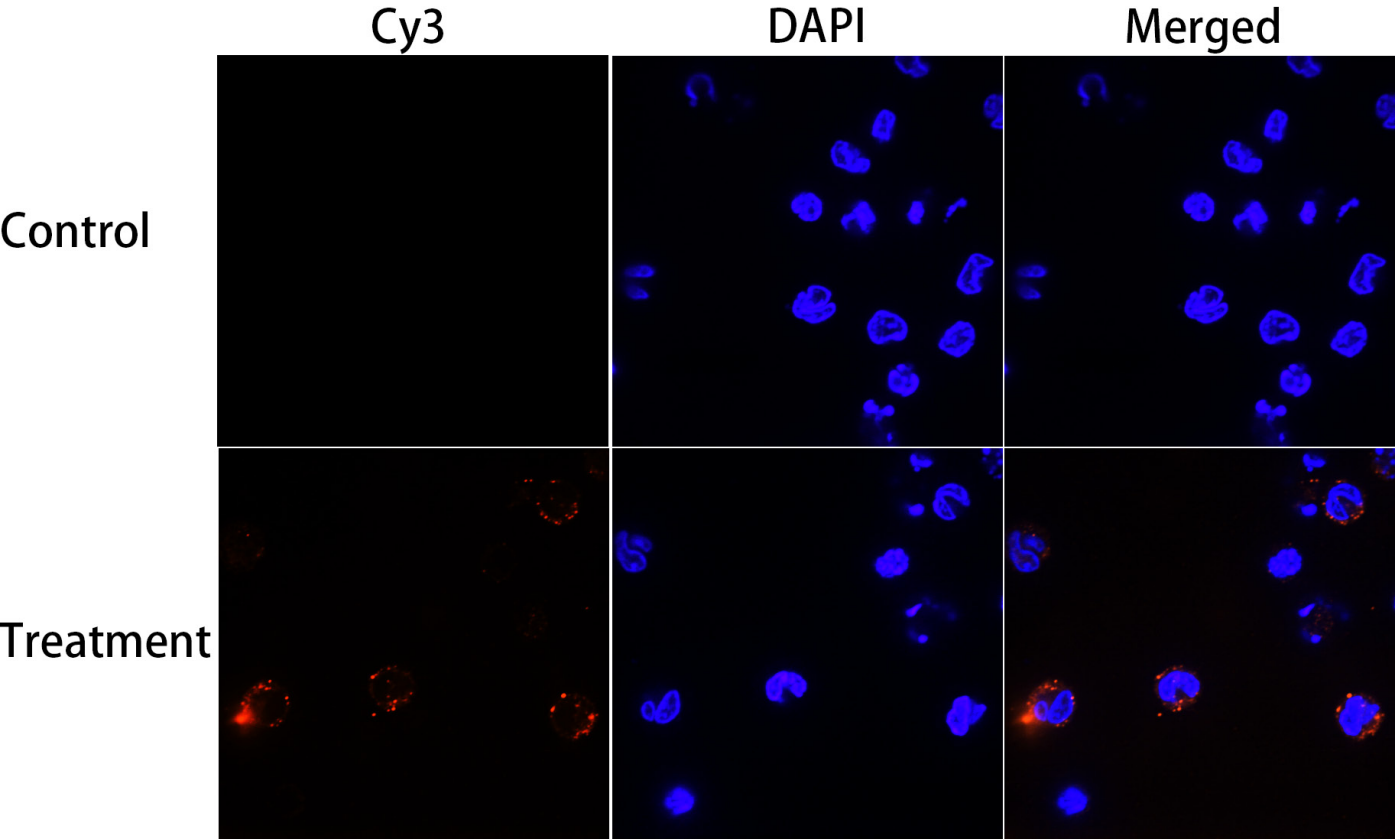
Binding of rHCMIF-1 to goat monocytes Goat monocytes were left untreated or incubated with rHCMIF-1 (40 μg/ml) for 1 h at 37°C. All cells were fixed and incubated with rat anti- rHCMIF-1 antibody followed by Cy3 labeled goat anti-rat IgG (red). The nuclei of the corresponding cells were visualized by DAPI (blue) staining. The internalizationofrHCMIF-1 by goat monocytes was visualized with a confocal laser scanning microscopy. Merge overlap of red and blue channels. The data are representative of three independent experiments.

Goat monocytes were incubated with rHCMIF-1 and the protein bind by monocytes was investigated by an immunofluorescence assay. As depicted in Figure 4, the emission from the Cy3-labeled rHCMIF-1 was red and the DAPI-labeled nuclei were blue. No fluorescence was observed under any color channel in the unstained background control (not shown). In the control group, no red fluorescence was observed (Figure 4 lower panel). Intense red fluorescence was observed when the cells were incubated with rHCMIF-1 (Figure 4 upper panel).

### The alteration of secreted cytokine levels

**Figure 5:**
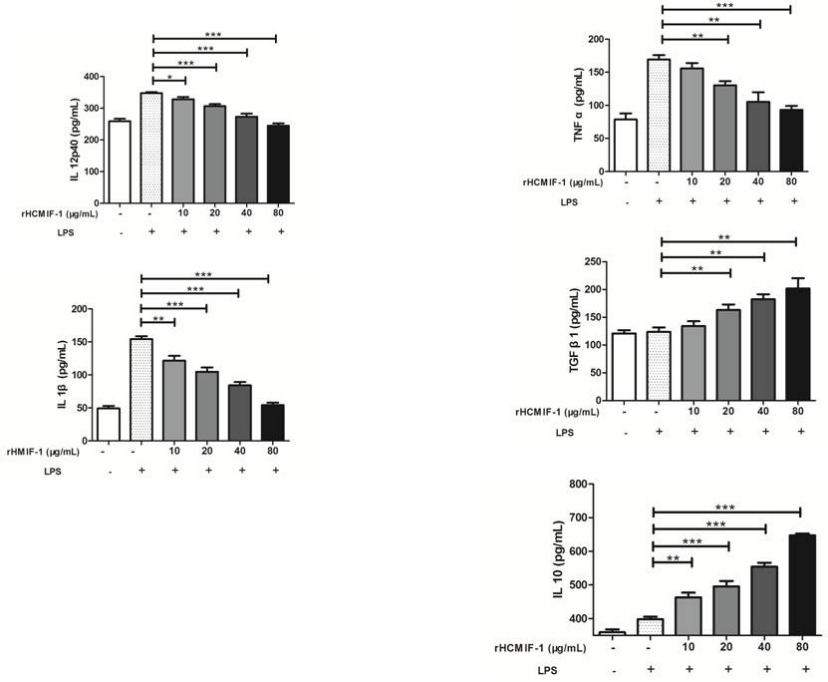
Regulation of cytokine secretion by rHCMIF-1 Goat monocytes were stimulated with LPS (100 ng/ml) for 72 h in the presence or absence of rHCMIF-1. Cytokine secretion in the supernatant of cell cultures was quantified by ELISA. The data are representative of three independent experiments (*p < 0.05, **p < 0.01, ***p < 0.001).

By performing ELISA we noted that rHCMIF-1 decreased the LPS induced production of TNF-α, IL-1β and IL-12p40 in goat monocytes. Intriguingly, rHCMIF-1 significantly increased the secretion of IL-10and TGF-β1 in goat monocytes in a dose-dependent manner compared with LPS treated only (Figure 5).

### rHCMIF-1 inhibited MHC-II expression on goat monocytes

Compared to the baseline expression of MHC-II in the control buffer, rHCMIF-1 significantly decreased MHC-II expression in a dose-dependent manner (Figure 7). However, no changes were detected in MHC-I following exposure of goat monocytes to rHCMIF-1 at different concentrations.

**Figure 6:**
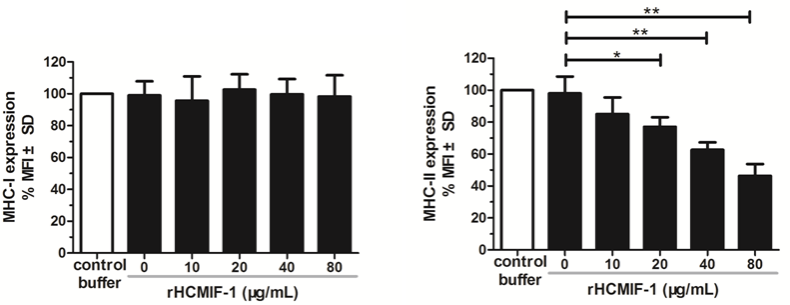
rHCMIF-1 inhibits MHC-II expression on goat monocytes Monocytes were cultured in the presence of control buffer (PBS/DTT) or different concentrations of rHCMIF-1for 24 h. MHC-II expression was measured by flowcytometry analysis and calculated as the percentage of mean fluorescence intensity (MFI) of controls. Bars represent the MFI ± SD of controls. (A) MHC-II (B) MHC-I. The data are representative of three independent experiments (*p < 0.05, **p < 0.01, ***p < 0.001).

**Figure 7:**
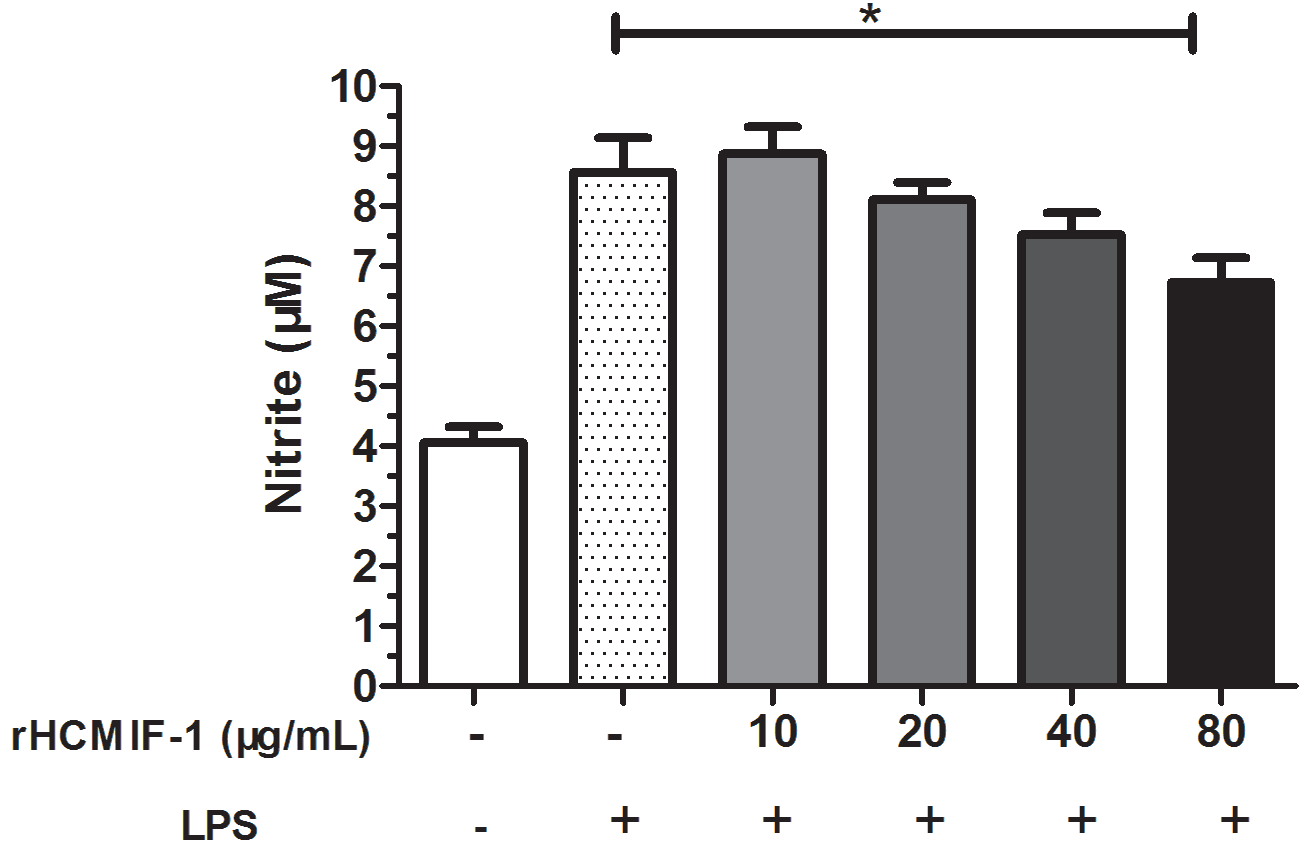
rHCMIF-1decreased NO production on LPS treated goat monocytes Monocytes were stimulated with LPS (100 ng/ml) for 48 h in the presence or absence of rHCMIF-1. NO was measured in the cell supernatants as nitrite using a NO assay kit. The data are representative of three independent experiments (*p < 0.05, **p < 0.01, ***p < 0.001).

### NO production

The nitrate concentration of the culture supernatant was significantly decreased by rHCMIF-1 in a dose-dependent manner. This suggested that rHCMIF-1 could down-regulate the LPS induced NO production of goat monocytes (Figure 7).

### Capacity of phagocytosis

**Figure 8:**
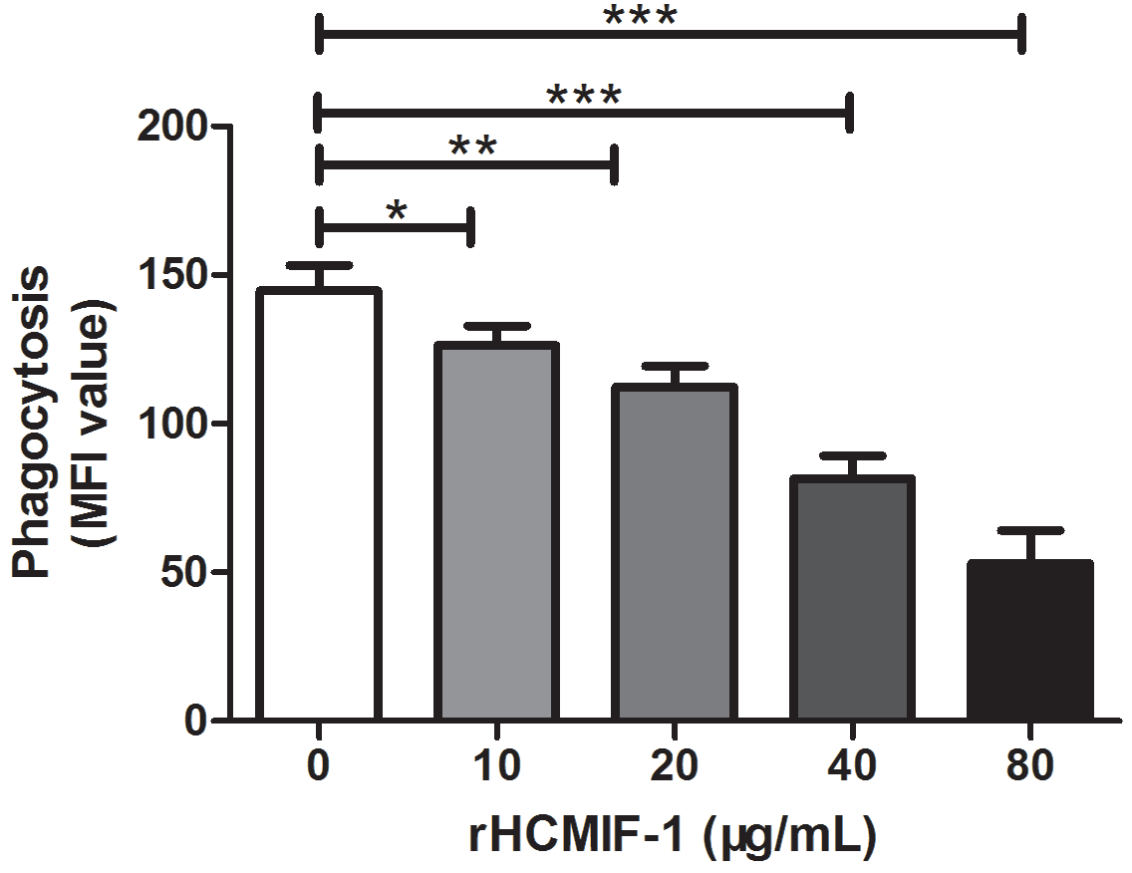
rHCMIF-1 decreased phagocytic capacity of goat monocytes Monocytes were collected after treated with rHCMIF-1 for 48 h and incubated with FITC-dextran (1mg/ml in RPMI1640) for 1 h at 37°C. The FITC-dextran internalization of cells was analyzed by flowcytometry and calculated as mean fluorescence intensity (MFI). The data are representative of three independent experiments(*p < 0.05, **p < 0.01, ***p < 0.001).

Phagocytic capacity of goat monocytes after 48 h treatment with different concentrations of rHCMIF-1 was examined. As shown in Figure 8, rHCMIF-1significantly decreased the FITC-dextran uptake ability of goat monocytes in a dose-dependent manner.

## DISCUSSION

The capacity of helminths parasites to modulate the immune system underpins their longevity in the mammalian host [25].There are several reports to show that nematode parasites that dwell in the gastrointestinal tract of their hosts are able to modulate the immune response systemically [26, 27].

In the present study a MIF homologue was described from the parasitic nematode *H. contortus* for the first time. A recombinant version of HCMIF-1 was produced, and enzymatic assays indicated that this recombinant protein possessed tautomerase activity as has been observed previously in other MIF-like molecules. We found that rHCMIF-1could be recognized by the antiserum from goats experimentally infected with *H. contortus* and the native HCMIF-1 protein was predominantly localized at the body surface and internal surface of the parasite's gut. Helminths parasite MIFs play immune modulating function, can be based on a hypothesis that host monocyte/macrophage can bind parasite MIFs [24]. Further, we demonstrated that goat monocyte could bind rHCMIF-1 *in vitro*. All of these results indicated that MIF of *H. contortus* were excretory/secretory antigens and interacted with the host immune system during infection. Theoretically, the immunomodulatory functions proposed require constant secretion and certain concentrations of this molecule. However, how HCMIF-1 accumulates to the functional concentration *in vivo* and the real mechanism through which it works during natural infection of *H. contortus* should be further studied.

As well as possible roles for the possible roles of MIFs in nematode homeostasis and development, it was also considered that parasitic nematode species express adapted ancestral genes to mimic host cytokines to modulate the immune system [28]. Extensive work has suggested that MIFs may be potent activators of the innate immune system capable of inducing proinflammatory mechanisms [29, 30]. Nisbetet al. indicated that highly purified recombinant *Strongyloides ratti* MIF (rSra-MIF) was found to induce IL-10 but not TNF-α production by MNC, and importantly, in the presence or absence of the PMB, excluding an LPS effect. The effectiveness of PMB was confirmed by neutralization of the LPS-induced cytokines [31]. These results were correspond to the results of Park et al. [32], who reported that the IL-10 and TGF-β levels in the bronchoalveolar lavage fluid were significantly higher following the treatment with the recombinant *Anisakis simplex* MIF (rAs-MIF). Further more, TGF-β and IL-10 were found to be increased in the spleen and mesenteric lymph nodes from the rAS-MIF treated mice [33], but there was no effect on the levels of IFN-γ, IL-6 and IL-13. The rAs-MIF appears to ameliorate dextran sodiumsulphate-induced colitis, suggesting that MIF might be useful as a therapeutic agent for the treatment of intestinal inflammatory disease. The recombinant MIF from *B. malayi* was able to induce cytokine production in human monocytes including endogenous human MIF. This suggests a positive feedback loop might exist in parasite-stimulated Hu-MIF expression due to the presence of high levels of MIF creating a local or possibly systemic anti-inflammatory host environment [34]. In addition, the secretion of MIF at the site of infection by the helminths, which might induce production of endogenous host MIF, may lead to blockade of AP-1-dependent proinflammatory gene expression by binding the transcription factor Jun activation domain-binding protein 1 (Jab1) [35]. Our results showed that rHCMIF-1 decreased the LPS induced production of TNF-α, IL-1β and IL-12p40 in goat monocytes. However, rHCMIF-1 significantly increased the secretion of IL-10 and TGF-β in goat monocytes in a dose-dependent manner compared with LPS treated only. The cytokines profile modulated byrHCMIF-1 contributed to the induction of an anti-inflammatory environment which is in favour of the worm's survival.

MHC-II molecules are constitutively expressed on the surface of APCs, enabling them to present extracellular antigens and initiate the adaptive immune response [36].Activation of APCs increases MHC-II expression [37].In the present study, we noted that rHCMIF-1 was able to inhibit MHC-II expression on monocytes in a dose dependent manner (Figure 5). This may due to the “deactivation” of monocytes triggered by high amounts of IL-10 [38]. But, the real mechanisms need further investigations. The main function of MHC-I is to display intracellular proteins to cytotoxic T cells. No significant change of MHC-I expression was observed after exposure of rHCMIF-1 in the present study. It might be that *H. contortus* is a kind of extracellular parasite and rHCMIF-1 does not affect the endogenous antigen presentation pathway. However, further studies are required to identify the actual mechanisms responsible.

Nitric oxide is synthesized by many cell types involved in immunity and inflammation [39]. It plays an important role in the majority of parasitic infections including *H. contortus*, by mediating host protection through either direct parasite killing or by limiting parasite growth [40–42].In the present study, rHCMIF-1 significantly decreased the NO production by LPS treated goat monocytes.

Phagocytosis is an early and fundamental step for the effective clearance of disease causing agents. The phagocytotic function of phagocytes is an important indicator of the body's immune competence [43]. The ability to engulf and kill pathogens is considered as a major effector function of macrophages [44].In the present study, phagocytic capacity of goat monocytes after treatment with different concentrations ofrHCMIF-1 decreased in a dose-dependent manner.

In conclusion, our results showed that rHCMIF-1 could bind by goat monocytes and exerts its immunomodulatory effects on multiple aspects to facilitate the immune evasion of *H. contortus.* These findings provided insight into the interactive relationship between parasitic nematode MIFs and host monocytes. It also shed new light on the molecular mechanisms of helminthic immune evasion. These findings may represent potential target as immunoregulator, and will be helpful to elucidate the molecular basis of host-parasite interactions and search for new potential protein as vaccine and drug target candidate.

## MATERIALS AND METHODS

### Ethics statement

The experiment was conducted following the guidelines of the Animal Ethics Committee, Nanjing Agricultural University, China. All experimental protocols were approved by the Science and Technology Agency of Jiangsu Province. The approval ID is SYXK (SU) 2010-0005.

### Parasites and animals

*H. contortus* strain (designated Nanjing 2005) was originally obtained from Nanjing (Jiangsu Province, China) and maintained by serial passage in 3-6-month-old, helminths-free goats [45]. Third stage larvae (L3) used for challenge were cultured from the feces of the monospecifically infected goats at 26°C and stored in water at a concentration of 2500 larvae/ml at 4°C.

Local crossbred male goats (3-6month-old) from the teaching and research flock at Nanjing Agricultural University were housed indoors in pens containing six goats per pen. The male goats were fed hay and whole shelled corn and provided with water *ad libitum*. All goats were dewormed twice at 2 week intervals with levamisole (8 mg/kg body weight) orally at the time of housing to remove naturally acquired strongylid infection. After 2 weeks, a fecal sample from each goat was examined by microscope for helminths eggs, according to standard parasitological techniques. Goats exhibiting no eggs were used in the subsequent study and daily health observations were performed throughout the experiment.

SD rats (body weight ~150 g) were purchased from Experimental Animal Center of Jiangsu, PR China (Qualified Certificate:SCXK 2008-0004) and were raised in a sterilized room and fed sterilized food and water.

### Cloning of HCMIF-1 and bioinformatics analyses

Utilizing resources from online database, the open reading frame (ORF) of MIF-like gene (GenBank accession number:CDJ88729.1) was amplified by reverse transcription-polymerase chain reaction (RT-PCR) using designed specific primers (forward primer: 5′-CG*GGATCC*ATGCCGGTTTTCTCATTT-3′ and reverse primer: 5′-CG*AAGCTT*AGCGAAGGTCTTGCTATT-3′), in which the *BamH*I and *Hind*III restriction sites respectively, were introduced and are shown in italic here. Following ligation of the obtained RT-PCR product with the pMD19-T vector (Takara, Dalian, China) to form pMD-MIF, the MIF fragment was cleaved from pMD-MIF by *BamH*I and *Hind*III and subcloned into the corresponding sites of pET28a vector (Invitrogen, Carlsbad, CA, USA). The accuracy of the insertion in the resulting plasmid was confirmed by sequencing.

### Expression and purification of rHCMIF-1 in *Escherichia coli*

The expression of the recombinant fusion protein in *E. coli* BL-21 cells (DE3) was induced by Isopropyl-β-D-thiogalactopyranoside (IPTG) at a final concentration of 1mM for 6 h at 37°C in Luria-Bertini (LB) medium with kanamycin (50 μg/ml). The histidine-tagged fusion protein was purified from the supernatant of bacterial lysates using the His·Bind^®^128 Resin Chromatography kit (Novagen) according to the manufacturer's instructions, and dialyzed in phosphate buffered saline (PBS, pH 7.4) to remove imidazole. The empty pET28a was used for producing control histidine-tagged protein, which was expressed and purified identical to the procedure for the MIF-histidine-tagged fusion protein. The purity of the purified rHCMIF-1 was analyzed by 12% sodium dodecyl sulfate polyacrylamide gelelectrophoresis (SDS-PAGE) followed by Coomassie blue staining. Protein concentrations were determined by Bradford method. LPS was depleted from the rHCMIF-1 using Detoxi-Gel Affinity Pak prepacked columns (Thermo Fisher Scientific, Waltham, MA, USA). The concentrations of the recombinant proteins were equalized to 1 mg/ml prior to LAL assay. Endotoxin levels ofthe protein samples were measured by LAL gel clot assay using a Pyrosate^®^Kit (Cape Cod Inc., East Falmouth, MA, USA). The samples whose endotoxin content was less than the sensitivity of the Pyrosate kit (<1EU per 1 mg of the recombinant proteins) were collected for the subsequent experiments.

### Enzyme activity assays

Using the substrate L-dopachromemethyl ester, the tautomerase activity was assayed as previously described by Nguyen et al. and Bendrat et al. [46, 47]. Tautomerase activity using *p*-hydroxyphenylpyruvate as substrate was performed following the methods described by Swope et al. and Wilson et al. [48, 49]. All substrates were purchased from Sigma (St. Louis, MO, USA).

### Generation of polyclonal antibodies

The goat antisera used in western blot analyses were collected from five goats experimentally infected with *H. contortus*. The goats were raised in helminths-free conditions and then orally challenged with 5000 infective L3. One month later, the goat antisera were collected and stored at −70°C until use.

To generate polyclonal antibodies against rHCMIF-1, 0.3 mg of purified rHCMIF-1 was mixed with Freund's complete adjuvant (1:1) and injected into SD rats subcutaneously in multiple places, following the method described by Wang et al. [50]. After the first injection, rats were then boosted four times at 2-week intervals with the same dose. The sera containing specific anti-rHCMIF-1 antibodies were harvested 10 days following the last injection and the specific reactivity with rHCMIF-1 was checked by enzyme-linked immunosorbent assay (ELISA).

### Western blot analysis

Purified rHCMIF-1 (20 μg) was resolved on 12% SDS-PAGE and transferred to Hybond-C extra nitrocellulose membranes (Amersham Biosciences, UK). Non-specific binding sites were blocked by immersing the membranes in 5% skim milk in Tris-buffered saline (TBS) for 1 h at room temperature. The membranes were then washed 5 times (5 min each) in TBS containing 0.1% Tween-20 (TBST). Subsequently, the membranes were incubated with the primary antibodies (antiserum from goats experimentally infected with *H. contortus*) for 1 h at 37 °C (dilutions 1:100 in TBST). After being washed 5 times in TBST, the membranes were then incubated with HRP-conjugated rabbit anti-goat IgG (Sigma, St. Louis, MO, USA) for 1 h at 37 °C (diluted 1:2000 in TBST). Finally, the immunoreaction was visualized using freshly prepared diaminobenzidine (DAB, Sigma) as a chromogenic substrate after 5 min.

### Localization of HCMIF-1 by immunohistochemical study

Washed adult worms suspended in PBS were fixed in 4% formaldehyde-0.2% glutaraldehyde in PBS for 90 min and then immersed in TISSUE-TeK^®^ O.C.T. compound (SAKURA, USA). They were snap frozen in liquid nitrogen and stored at -20°C until required for further processing. Cryostat sections of 10 μm thickness were cut, washed with PBS and treated for 60 min with 10% normal goat serum in PBS to prevent non-specific binding of antibodies. The sections were then incubated with specific rat anti-rHCMIF-1 antiserum (1:100 dilution) or normal rat serum(control) for 60 min at 37°C, washed 15 min × 3 with PBS, and subsequently incubated for 60 min with Cy3 goat anti-rat IgG (ab6953, Abcam, Cambridge, MA, USA). Finally, the sections were stained with DAPI (Beyotime, Haimen, Jiangsu, China) to show DNA. After washing with PBS, the specimens were immersed in Anti-Fade Fluoromount solution (Beyotime), which prevents fading of fluorescence during microscopic examination.

### Isolation of goat monocytes

Peripheral blood mononuclear cells (PBMCs) were separated from heparinized blood with the standard Ficoll-hypaque (GE Healthcare, USA) gradient centrifugation method and washed twice in PBS. Monocytes were isolated by their adherence to plastic surface [51]. The goat PBMCs were seeded in a 6 wells flat-bottom tissue culture plates (Corning, USA) in cell culture medium RPMI 1640 (GIBCO, UK) containing 10% heat inactivated fetal calf serum (GIBCO, UK), 100 U/mL penicillin and 100 mg/mL streptomycin (GIBCO, UK). Plates were incubated at 37°C in a humidified atmosphere with 5% CO2 for 1 h [52]. Non-adherent cells were removed by washing twice with PBS. The adherent cells were collected and adjusted to a density of 1×10^6^ cells/mL in cell medium at 37°C in a humidified atmosphere with 5% CO2. Cell viability, as determined by trypanblue dye exclusion, was more than 95% in all cases.

### Binding of rHCMIF-1 to goat monocytes

Freshly isolated goat monocyte as described above were seeded into 24-well plates and cultured on glass coverslips with rHCMIF-1 (40μg/ml). The non-treated cells were set as control. After incubation at 37 °C for 1 h, the coverslips were washed with 0.1 M PBS and fixed in 4% paraformaldehyde for 30 min at room temperature. The cells were then incubated with PBS containing 5% normal goat serum for 30 min at room temperature to block non-specific binding before the addition of rat anti-rHCMIF-1 polyclonal antibodies (diluted 1:100 in PBS containing 5% normal goat serum), which was incubated overnight at 4 °C. Primary antibodies were detected by Cy3 goat anti-rat IgG (ab6953, Abcam) diluted 1:400 with the same blocking solution and incubated at 37 °C for 1 h. Cells were counterstained with DAPI (Beyotime Institute of Biotechnology, China). Thus, rHCMIF-1 and nucleus appeared to be red and blue, respectively. The cells were visualized with a confocal laser scanning microscopy (LSM 710; Carl Zeiss, Germany) with a 100× oil immersion objective. Red and blue color channels, corresponding to Cy3 and DAPI, respectively, were selected to acquire image. The fluorescence settings used for rHCMIF-1-treated cells were the same to that used for control cells. An unstained control was conducted to detect auto-fluorescence or background staining of the cells and the protein. Image acquisition and synergistic combination were performed automatically using ZEN software (Carl Zeiss, Germany). Three independent samples were investigated.

### Detection of cytokine secretion

To determine cytokine secretion, goat monocytes were stimulated with LPS (100 ng/ml) for 72 h in the presence or absence of rHCMIF-1. The supernatants were collected and cytokine testing was performed by ELISA. The levels of TNF-α, IL-1β, IL-10, IL-12p40 and TGF-β1 in supernatants were determined using commercially available goat ELISA kits (Anoric, Tianjin, China). The analysis was performed with the data from three independent experiments.

### Analysis of MHC molecule expression

The purified monocytes (0.5×10^6^ cells/ml) were incubated with different concentrations of rHCMIF-1 or equal volumes of control buffer for 24 h in complete RPMI 1640 at 37 °C. Cells were then stained with the monoclonal antibodies to MHC-I (MCA2189A647, AbDserotec, BioRad Laboratories, CA, USA) and MHC-II (MCA2226F, AbDserotec), and analyzed on a FACS Calibur cytometer (BD Biosciences, San Jose, CA, USA). Results were expressed as the percentage of mean fluorescence intensity (MFI) of control.

### Measurement of nitric oxide production

To determine nitric oxide production, goat monocytes were stimulated with LPS (100 ng/ml) for 48 h in the presence or absence of rHCMIF-1. NO was measured in the cell supernatants as nitrite using a NO assay kit (Beyotime) according to the manufacturer's protocol. Briefly, a standard curve was prepared with standard nitrite solutions in DMEM medium. The standard solutions or cell supernatants were reacted with nitrate reductase for 30 min in a 96-well plate, and then Griess reagent I and Griess reagent II were added. After 10 min incubation at room temperature, the absorbance at 540 nm was read in a microplate reader (Bio-Rad, Hercules, CA, USA). The samples were assayed in triplicate.

### FITC-dextran internalization

To confirm the effect of rHCMIF-1on the phagocytotic ability of goat monocytes, the FITC-dextran internalization of cells was analyzed by flowcytometry. Cells were collected after treated with rHCMIF-1 for 48 h and incubated with FITC-dextran (1mg/mlin RPMI1640) for 1 h at 37°C. Cells added with the same amount of FITC-Dextran and incubated at 4°C for 1h were used as the baseline of monocyte phagocytosis. After incubation, cells were washed extensively to remove excess FITC-dextran. The FITC-dextran internalization of cells was analyzed by flowcytometry (BD Biosciences) using Cell QuestSoftware and median fluorescence intensity (MFI) was calculated.

### Statistical analysis

Data are expressed as mean ± the standard deviation of the mean. Statistical analysis for significant differences was performed using an analysis of variance, the Student's *t* test for parametric samples (GraphPad Prism, USA).
